# Rotation Error Prediction of CNC Spindle Based on Short-Time Fourier Transform of Vibration Sensor Signals and Improved Weighted Residual Network

**DOI:** 10.3390/s24134244

**Published:** 2024-06-29

**Authors:** Lin Song, Jianying Tan

**Affiliations:** School of Intelligent Manufacturing, Panzhihua University, Panzhihua 617000, China

**Keywords:** spindle, rotation error, prediction, weighted, attention, ResNet, STFT

## Abstract

The spindle rotation error of computer numerical control (CNC) equipment directly reflects the machining quality of the workpiece and is a key indicator reflecting the performance and reliability of CNC equipment. Existing rotation error prediction methods do not consider the importance of different sensor data. This study developed an adaptive weighted deep residual network (ResNet) for predicting spindle rotation errors, thereby establishing accurate mapping between easily obtainable vibration information and difficult-to-obtain rotation errors. Firstly, multi-sensor data are collected by a vibration sensor, and Short-time Fourier Transform (STFT) is adopted to extract the feature information in the original data. Then, an adaptive feature recalibration unit with residual connection is constructed based on the attention weighting operation. By stacking multiple residual blocks and attention weighting units, the data of different channels are adaptively weighted to highlight important information and suppress redundancy information. The weight visualization results indicate that the adaptive weighted ResNet (AWResNet) can learn a set of weights for channel recalibration. The comparison results indicate that AWResNet has higher prediction accuracy than other deep learning models and can be used for spindle rotation error prediction.

## 1. Introduction

High-precision computer numerical control (CNC) equipment is the core of the modern manufacturing industry. The spindle is the key rotating part of CNC equipment, which is a complex mechanical system integrating machine, electricity, liquid and gas. Rotation error refers to the distance that the actual rotation axis deviates from its ideal axis [1]. The spindle rotation error of CNC equipment directly reflects the machining quality of the workpiece and is a key indicator reflecting the performance and reliability of CNC equipment [2,3]. Accurately predicting the rotation error of the spindle is of great significance for reducing machining errors and improving the reliability of CNC equipment.

Through reviewing the existing literature, it is found that spindle rotation error prediction is mainly divided into the direct measurement method and the physical modeling-based method. The direct measurement method installs a standard ball or rod at the end of the tool jig, and the rotation accuracy tester is utilized to measure the spindle rotation error. Based on the direct measurement method, researchers have conducted extensive research on monitoring spindle rotation errors. For example, Castro [4] proposed a laser interferometer-based method for evaluating the rotation error of machine tool spindles, utilizing a master ball with high surface finish and accuracy to reflect the incident beam back to the interferometer. Liu et al. [5] proposed a four-point method for spindle rotation error measurement and separation by using four sensors to measure the orbit at the center of the spindle cross-section. Wang et al. [6,7] developed a spindle rotation error evaluation method based on the least squares method, which is based on a measuring system composed of a standard ball and high-precision capacitive displacement sensor. The error characteristics are extracted by time domain and frequency domain signal analyses. At the same time, it was found that the rotation error is closely related to the spindle rotation speed. Anandan et al. [8] proposed a multi-directional error separation technique to obtain the radial axis rotation error.

The physical modeling-based approaches carry out spindle vibration analysis and rotation error prediction by establishing a spindle dynamics model. For example, Karacay et al. [9] studied the spindle vibration in radial, axial, rocking and yawing directions, utilizing a model of spindle dynamics of a rigid rotor grinder supported by angular contact ball bearings. Kang et al. [10] developed a physical model of a high-fidelity and high-speed spindle bearing system and realized the dynamic prediction of spindle rotation error. Bai [11] studied the formation mechanism of rotation error and concluded that the bearing, spindle and spindle shank joint face are the key components that lead to the decline in spindle rotation accuracy, and they established a physical model of spindle rotation accuracy degradation based on bearing wear.

The above review shows that direct measurement methods and physical model-based methods are able to obtain the spindle rotation error. However, there are still some limitations to these methods. The main drawback of direct measurement methods is that the tool position is occupied by a standard ball or standard bar. The spindle being measured cannot mount the tool and complete the normal machining process [12]. Therefore, the direct measurement methods are based on the premise of an idle spindle and cannot measure the rotation error when the spindle is loaded [13], which is challenging to use in the actual machining of workpieces with cutting tools. Most current studies utilize the spindle dynamics model to study the spindle stiffness, intrinsic frequency and other dynamics parameters, and carry out the optimization design. Due to the extensive simplification of the rolling bearing dynamics model, few studies have predicted the spindle rotation error [10]. In addition, establishing a dynamic prediction model is an extraordinarily time-consuming and idealized process, which is not conducive to industrial practical applications. The real-time monitoring of machine tool spindle performance and the real-time compensation of rotation errors have become enormous challenges. Complex and simplified dynamic models do not accurately reflect spindle rotation; expensive measurement equipment, strict installation requirements and existing measurement techniques can affect regular machining tasks. These challenges create obstacles to the direct prediction of spindle rotation errors.

According to references [6,7,11], the spindle rotation error is closely related to speed and wear degree. Spindle vibration signals usually contain characteristic information about spindle speed [14] and wear level [15]. Therefore, it is reasonable and feasible to establish the function mapping relationship between the spindle rotation error and vibration signal [16]. In fact, the realization of rotating machinery fault diagnosis [17,18,19] and remaining useful life prediction [20,21,22] through vibration signals has been rapidly developed. The difference with fault diagnosis is that the spindle rotation error is a non-fault state, the vibration signals are similar between categories and the discriminative features are weak, unlike the significant difference in features between different fault categories. Spindle rotation error prediction is also not a regression problem, such as remaining useful life prediction, and the regression method is not generalized enough for rotation error prediction at multiple speeds [12]. With the help of the above idea, researchers established a mapping relationship between the vibration signal and the rotation error by means of a neural network. Song et al. [23] developed a multi-scale convolutional neural network (MSCNN) model for spindle rotation error prediction by first acquiring spindle vibration signals through multiple sensors and then extracting features using convolutional kernels of different sizes. The experimental results verify the feasibility of the prediction of spindle rotation error by a convolutional neural network (CNN). Further, to address the bottleneck regarding traditional CNN models, which are difficult to train when superimposing deep structures, Song et al. [24] proposed a residual network (ResNet)-based spindle rotation error prediction algorithm, which achieved good prediction accuracy. However, existing CNN-based methods do not consider the correlation of multi-sensor data, affecting the accuracy of deep learning methods. Specifically, due to different installation locations, data from different sensors may contain various degrees of degraded information. In particular, data collected by multiple sensors are redundant, and the direct fusion of different channels without distinguishing the importance of the sensors may lead to the transfer of redundant information between networks, further affecting the performance of the model. So, deep learning requires effective learning mechanisms to highlight sensor data that contain more degraded information to improve the generalization ability of the model. The categories for spindle rotation error prediction are non-fault states, and the small intra-class distance further increases the difficulty of prediction.

To address the above problems, this article developed a new neural network model named adaptive weighted ResNet (AWResNet) for predicting spindle rotation error. The method incorporates an adaptive multi-sensor data-recalibration module in ResNet to weight the channel data, thus improving the accuracy of spindle rotation error prediction. In order to assess the effectiveness and superiority of the developed model, spindle rotation error prediction experiments were carried out. The main contributions of this article can be summarized as follows:

1. The attention weighting unit is adopted to adaptively distinguish the importance of the spindle multi-sensor vibration data, so as to emphasize the important feature information, suppress the redundant feature information, and enhance the feature extraction capability of the model.
2. The AWResNet model for spindle rotation error prediction is constructed by adding an attention weighting unit to the original residual network (ResNet), which takes the Short-time Fourier Transform (STFT) time-frequency domain features of the vibration signals as inputs to establishes end-to-end mapping between the vibration signals and the rotation errors.
3. Comparison tests, feature visualization, attention weight visualization, and anti-noise experiments are carried out based on the vibration data collected from the machine tool spindle reliability test bed, and the experimental results verify the effectiveness and superiority of the proposed method.

The rest of the article consists of Section 2, which introduces the fundamentals of ResNet; Section 3, which describes in detail the developed AWResNet prediction model; Section 4, which employs the spindle rotation error dataset to verify the validity and superiority of AWResNet; and Section 5, which draws conclusions.

## 2. The Fundamentals of ResNet

### 2.1. Convolution Neural Network

The CNN [25,26] is an important branch of deep learning methods. Because of its strong feature-learning ability, it has been widely used in the manufacturing industry in equipment reliability analysis fields such as fault diagnosis [27,28], remaining useful life prediction [29,30] and so on. In the CNN model, a trainable convolution kernel slides over the input data to extract local features at different positions. Sparse connection and weight sharing are the main features that distinguish CNNs from traditional neural networks. Convolutional operations are performed by multiplying the convolution kernel with the corresponding positions of the input data and then adding them to obtain the output [31]. The convolution operation can be expressed as follows:

(1) $$x_{j}^{l}=f(\sum x_{i}^{l-1}*\omega_{ij}^{l}+b_{j}^{l})$$

where x indicates the output of the convolution layer; ω indicates the weight of the convolution kernel and b is the bias; i, j and l represent the serial numbers of input channels, output channels and the convolution layer, respectively; ∗ represents convolution operation; f(·) represents the nonlinear transformation activation function. The derivative of the rectified linear unit (ReLU) can only be 0 or 1, which is more effective than the traditional Sigmoid and Tanh activation functions at avoiding gradient disappearance and gradient burst during deep neural network training.

### 2.2. Pooling

**Figure 1:**
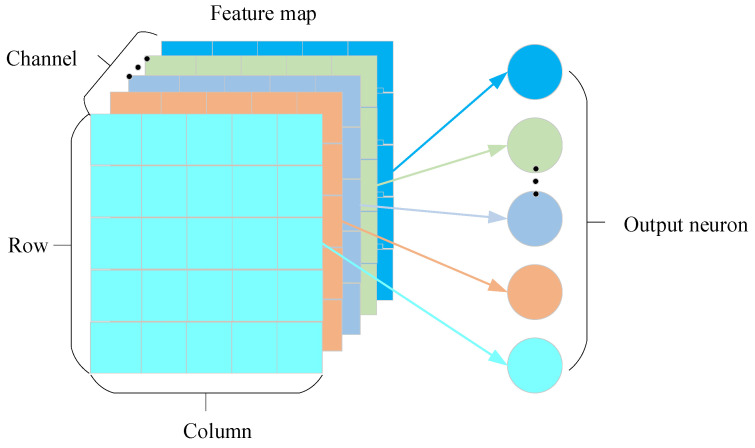
GAP schematic diagram.

Pooling is a downsampling operation that significantly compresses the data and reduces the data dimension. The specific operation of the pooling layer is to aggregate a data point with its surrounding data points to reduce the data dimensions. Commonly used pooling operations are average pooling and maximum pooling. Average pooling takes the average value of the data points in a specific region of the feature map in a particular step size, while maximum pooling takes its maximum value. Taking maximum pooling as an example, its mathematical description can be expressed as follows:

(2) $$p_{c}\left(k,z\right)=max\{x_{c}(m+s\left(k-1\right),n+s\left(z-1\right))\}$$

where $p_{c}\left(k,z\right)$ is the output at coordinates (c,k,z); $x_{c}\left(k,z\right)$ is the input data at c-th channel, k-th row and z-th column, where m, n∈[1, i]; i represents the size of the pooling region; s represents the sampling interval. Global average pooling (GAP) is shown in Figure 1, which takes the average of all the data in each channel and is mainly used before the fully connected layer of the ResNet model, thus enabling data dimensionality reduction.

### 2.3. Cross-Entropy Loss Function

The loss function for classification tasks is generally cross-entropy loss (CEL). In the classification calculation, the estimated probability $q_{i}(y)$ that the observation y belongs to class i can be calculated and compared with the true probability $p_{i}(y)$ for obtaining the loss of the CNN. In deep learning methods, CEL calculates the distance between the predicted and true values. CEL can be expressed as follows:

(3) $$L=-\sum_{i=1}^{M}p_{i}(y)log(q_{i}(y))$$

In the CEL function, M represents the number of classification categories.

## 3. The Proposed Prediction Method

The existing spindle rotation error tester is expensive and requires high installation accuracy, and it is easy to cause damage in practical applications. Therefore, it is challenging to measure rotation error under load. How to monitor the performance of the spindle and compensate for the error in real time has become an urgent problem. The prediction method based on vibration signal does not consider the importance of different sensor data. To solve the above issues, this section adopts the original vibration signal as the input and firstly extracts the time–frequency characteristics of the original multi-sensor data by using STFT. Then, in order to distinguish the importance of different sensor data and establish the correlation of multi-sensor data, a new AWResNet method is proposed to adaptively recalibrate different sensor data to give more weight to data containing more degenerate information and less weight to data containing redundant information, so as to extract more discriminative features and improve the prediction ability of deep learning networks.

### 3.1. STFT Representation

**Figure 2:**
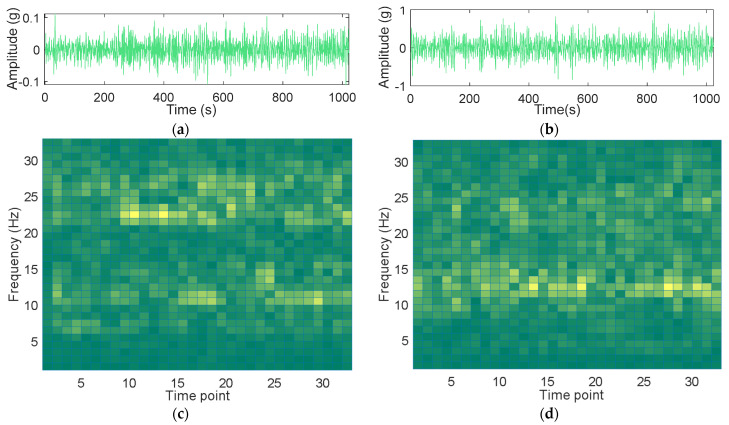
Time and time-frequency domain characterization of vibration data: (**a**) Time domain representation of spindle-end vibration signal with a rotation speed of 1000 r/min and a rotation error of 8.5 μm; (**b**) time domain representation of spindle-end vibration signal with a rotation speed of 3000 r/min and a rotation error of 12 μm; (**c**) time–frequency domain representation of spindle-end vibration signal with a rotation speed of 1000 r/min and a rotation error of 8.5 μm; (**d**) time-frequency domain representation of spindle-end vibration signal with a rotation speed of 3000 r/min and a rotation error of 12 μm.

STFT is a joint time–frequency transform method for non-stationary signals. It converts one-dimensional vibration signals into a two-dimensional matrix suitable for two-dimensional CNN processing, which contains not only the time domain characteristic spectrum, but also the frequency domain characteristic spectrum. STFT divides the original vibration data into equal-length segments, then multiplies each segment by a window function in chronological order to perform a segmented Fourier transform. The results of the obtained series of Fourier transform are lined up to become a two-dimensional representation. Mathematically, STFT can be written as follows:

(4) $$STFT(t,\omega )=\int_{-\infty }^{+\infty }x(\tau )h(\tau -t)e^{-j\omega t}d\tau$$

where t and τ are time; ω is frequency; x(τ) is the signal that needs to be transformed; h(τ−t) is the window function; and STFT(t,ω) is the Fourier transform of x(τ)h(τ−t). This paper adopts the Hann window as the window function, and the Hann window function can be expressed as follows:

(5) $$h\left(\tau \right)=0.5-0.5\cos\left(\frac{2\pi \tau }{K-1}\right)$$

where K denotes the number of data points in the output of each Fourier transform segment, which is set to 64 in this article. An input signal of length 1024 is passed through a Hann window STFT with an overlap of 32 to obtain a two-dimensional representation of 33×33. Figure 2 illustrates the raw vibration data and their corresponding STFT time-frequency representation. (a) and (b) represent the time domain of the vibration sensor at the spindle end with a rotation error of 8.5 µm when the spindle speed is 1000 r/min and the vibration sensor at the bearing end with a rotation error of 12 μm when the spindle speed is 3000 r/min, respectively. (c) and (d) are time–frequency domain representations of (a) and (b), respectively.

By observing the time domain signals under different rotation errors, it is found that the vibration data under different rotation errors have apparent similarities. In the STFT time-frequency domain feature map, the vertical axis indicates the frequency bands at different frequencies, and the horizontal axis indicates the time points. It can be seen that researchers find it difficult to simply use time or time-frequency domain signal analysis methods to distinguish different categories of rotation errors.

### 3.2. The Proposed AWResNet Model

**Figure 3:**
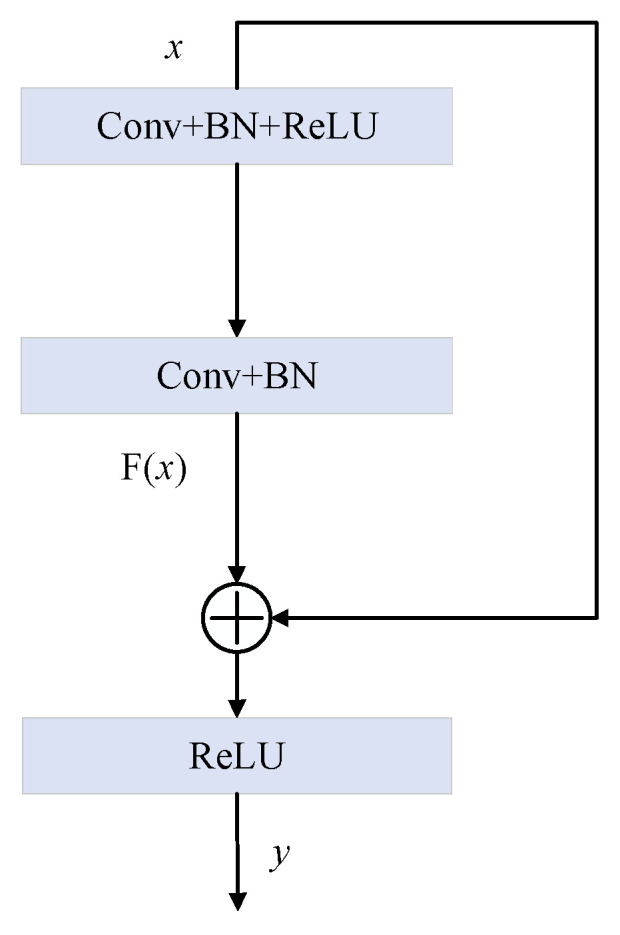
Residual building unit.

ResNet was developed to solve the problem of deep CNN training in image processing. The ResNet model adds many identity maps to the convolution layer, which is beneficial to the backpropagation of errors and optimization of network weights. ResNet has performed well in image recognition, image segmentation and object detection [32]. Figure 3 shows the residual building units (RBUs) of ResNet. Each RBU module consists of Conv, BN [33] and ReLU. Stacking multiple RBU modules builds the ResNet model. In Figure 3, Conv represents the convolutional layer. The output y of the entire RBU can be represented as follows:

(6) y=ReLU(F(x)+x)

where BN stands for Batch Normalization. When the batch training method is used, the feature distribution among samples often changes during iteration, which is an internal covariance shift problem. In this case, the model parameters need to be constantly updated to accommodate the changing distribution. BN is a normalization method to solve the problem of internal covariance shift.

#### 3.2.1. Attention Weighting Unit

**Figure 4:**
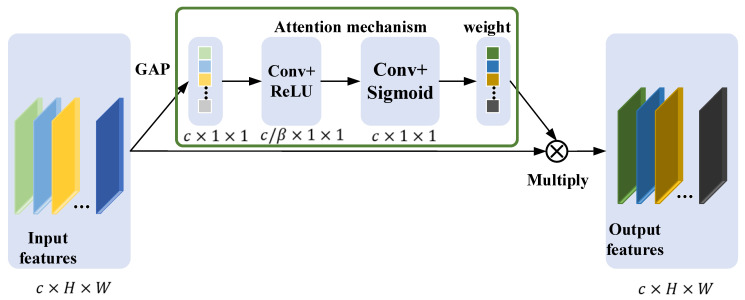
Attention weighting unit.

The STFT time-frequency representation of the multi-sensor data is utilized as an input to the ResNet model, with each input data channel representing a sensor signal. Data from different sensors contain information about spindle degradation to varying degrees. Specifically, some data may contain rich feature information related to spindle degradation features, while others may contain very few degradation features or even only measurement noise. Therefore, in order to identify discriminatively important information in multi-sensor data, it is necessary to identify the importance of different sensor data by modeling the relationship between channels along the channel dimension. To highlight the critical feature information and suppress the useless feature information, an adaptive weighting ResNet (AWResNet) model is proposed for the recalibration of spindle multi-sensor data. The core idea of AWResNet is to add the squeeze-excitation attention weighting unit [34] to the RBU. The attention weighting unit can adaptively recalibrate the weights of multi-sensor channel data according to the input, with each channel’s data having a weight of varying magnitude. A larger weight represents the greater influence of the channel’s data, with vice versa indicating that the channel’s data are less important. The attention weighting unit is displayed in Figure 4 and comprises four parts: global information extraction, channel interrelationship modeling, weight calculation and weighted output.

Global information extraction: In order to establish dependencies between different channel information, it is necessary to compress global spatial information into channel descriptions, which is achieved through GAP operations. The global information of statistical information z in channel c can be represented as follows:

(7) $$z_{c}=\frac{1}{H\times W}\sum_{k=1}^{H}\sum_{z=1}^{W}x_{c}(k,z)$$

where $x\in R^{c\times H\times W}$, H×W represents the spatial dimension of $x_{c}$, and the input signal is an STFT time-frequency representation with a height of H and a width of W. $z\in R^{c\times 1\times 1}$, $z_{c}$ can be interpreted as a channel description, which can describe the global information of different channels. $x_{c}$ represents the input feature information in the c-th channel.

Channel inter-relationship modeling: To establish the inter-relationships between channels and capture the dependencies between different channel information, this step must be able to learn the nonlinear interactions between different channels, which is implemented through a one-dimensional convolutional Conv and ReLU activation function. The input for this step is $z\in R^{c\times 1\times 1}$, with a channel of c. The convolution operation with a kernel of 1×1 can fuse information from different channels. In order to control the computational complexity of the attention mechanism, the number of output channels of the one-dimensional convolution is reduced by the dimension parameter β. The number of input channels of the one-dimensional convolution is c, and the output channels is c/β. Feature nonlinear transformations are implemented by employing ReLU after one-dimensional convolution.

Weight calculation: The weight calculation must ensure that the feature information of multiple channels is allowed to be emphasized rather than a single channel, and the weight of each channel is obtained by adopting the Sigmoid function. The output value of the Sigmoid is between 0 and 1, which ensures that multiple channels are emphasized. Before activating the Sigmoid function, it is also necessary to increase the dimension through a one-dimensional Conv with a convolution kernel of 1×1, so that the number of weights is consistent with the number of channels. The convolutional layer has c/β input channels and c output channels. The process of channel relationship modeling and weight calculation can be expressed as follows:

(8) $$s=\sigma (W_{2}\delta (W_{1}z))$$

where σ represents Sigmoid and δ represents ReLU function; s denotes attention weight; the weight of the first convolutional layer is $W_{1}$, and the second convolutional layer is $W_{2}$. It should be noted that the value of attention weight s will adaptively change with the input sample. By adopting the channel attention mechanism, different samples can adaptively learn a set of weights of different sizes, thereby assigning larger weights to more important channels and smaller weights to less important channels.

Weighted output: Multiply the input feature x and attention weight to obtain a weighted output, and the final recalibrated output ${\bar{x}}_{c}$ can be expressed as follows:

(9) $${\bar{x}}_{c}=s_{c}x_{c}$$

#### 3.2.2. AWResNet Model

**Figure 5:**
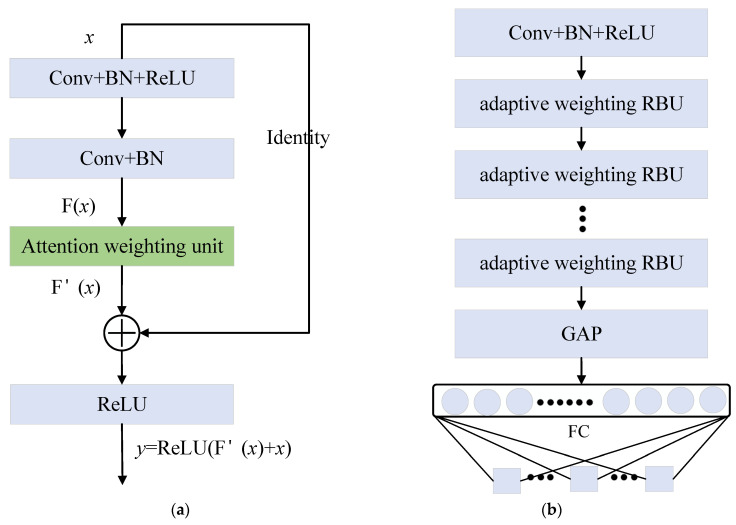
Adaptive weighting RBU and AWResNet model: (**a**) Adaptive weighting RBU; (**b**) AWResNet model.

By embedding an attention weighting unit in the RBU module, the adaptive weighting of data from different channels can be realized. Figure 5a shows the proposed adaptive weighting RBU module. The attention weighting unit is located after the second BN layer. The proposed AWResNet model can be constructed by stacking multiple adaptive weighting RBU modules, as shown in Figure 5b, where FC indicates the fully connected layer.

Based on the AWResNet model proposed above, the model’s parameters need to be further determined. The structure of AWResNet is illustrated in Table 1. In the table, adaptive weighting RBU1 ×2 represents the residual block repeated once. Therefore, the structure contains 17 convolution and 1 fully connected layer operations. In adaptive weighting RBU, the dimension parameter β is set to 16.

#### 3.2.3. AWResNet-Based Spindle Rotation Error Prediction Procedure

**Figure 6:**
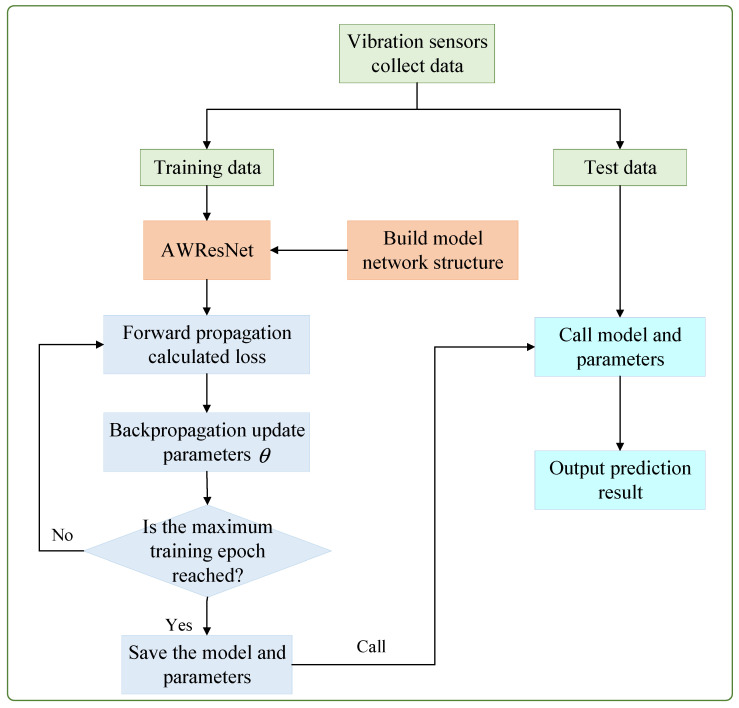
The spindle rotation error prediction procedure of the proposed AWResNet method.

Figure 6 shows the AWResNet-based spindle rotation error prediction procedure, which can be summarized into four steps: data collection and preprocessing, network structure design, model offline training, and model online testing. The detailed steps are as follows:

(1) Data collection and preprocessing. Firstly, multiple vibration sensors were employed to acquire vibration data from the spindle with different rotation errors. Secondly, the vibration signals were divided by a fixed length, and corresponding labels were made for each divided sample according to the category of rotation error. Meanwhile, STFT processing converted the signal into time–frequency domain features. Then, min–max normalization was used to preprocess the data, which is conducive to the convergence of the model.

(2) Network structure design. The network structure adopted in this paper is shown in Table 1.

(3) Model offline training. The prepared training set data were fed into the AWResNet model, and the model was trained by the gradient descent method. Forward propagation calculated the loss, and back propagation updated the model parameters. The detailed algorithm flow for training AWResNet model is presented in Algorithm 1. When the maximum training epoch was reached, the trained model weight and bias were saved as local files for model testing and calling during online deployment.

(4) Model online testing. The saved AWResNet model structure and parameters were called, the prepared test set was taken as the input of the trained AWResNet algorithm, the actual classification probability was calculated, and the prediction result of the rotation error was obtained.
**Algorithm 1:** AWResNet training procedure**Input:** Training dataset: $D={\left\{x_{i},y_{i}\right\}}_{i=1}^{n}$; Learning rate: η; Maximum training epoch: E.
1: **for** epoch = 1, 2, 3, ……, E
**do**
2:  //Feature extract 3:  Calculate the output of Conv+BN+ReLU layers; 4:  Calculate the output of 8 adaptive weighting RBU modules in series; 5:  Calculate the output of the GAP layer; 6:  Calculate the output $x_{i}$ of the FC layer; 7:  //Calculate the probability $p_{j}$ of each category 8:  $p_{j}=\frac{e^{x_{j}}}{\sum_{i=1}^{M}e^{x_{i}}}$, where M stands for the number of categories; 9:  //Calculate loss 10:   Calculate the cross entropy loss L(p(x),q(x)) using Formula (3); 11:   //Error backpropagation and updating parameters 12:   $W^{*}\leftarrow W-\eta (\frac{\partial L}{\partial W})$, $b^{*}\leftarrow b-\eta (\frac{\partial L}{\partial b})$ 13: **end for** **Output:** $\theta^{*}=\left\{W^{*},b^{*}\right\}$


## 4. Experimental Verifications

To evaluate the effectiveness and superiority of the developed AWResNet model in predicting the rotation error of spindle of CNC machines, vibration data and the corresponding rotation error were collected through a spindle reliability test bed. Python 3.7 and PyTorch was adopted as programming language to carry out experimental verification and analysis in the hardware environment of Windows, i7 CPU and RTX2060 SUPER GPU. It should be noted that to ensure the fairness of the comparison results, the experimental results of all methods were obtained under the same data acquisition platform, the same dataset and the same programming environment.

### 4.1. Experimental Platform

**Figure 7:**
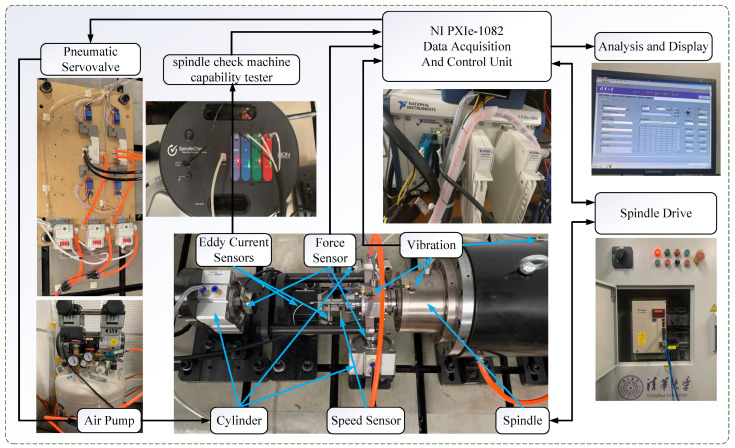
Test bench for spindle rotation error.

Figure 7 illustrates the spindle rotation error reliability analysis testbed, which consists of a CTB40D spindle and drive, a PCB256A14 vibration sensor, a DYMH-104 force sensor, a rotation signal processing unit, an NI PXie-1082 data acquisition and control unit, etc. The experimental platform can collect vibration signals and rotation signals through the vibration sensor and spindle rotation signal processing unit, respectively. The vibration sensors are installed at the base end, spindle end and bearing end. The loading experiments were performed through the load spectrum of the spindle to ensure that the spindle operation was similar to the actual working conditions [35]. The rotation signal processing unit collected the rotation error signal every 10 h and performed wear tests at other times.

### 4.2. Data Preprocessing

**Figure 8:**
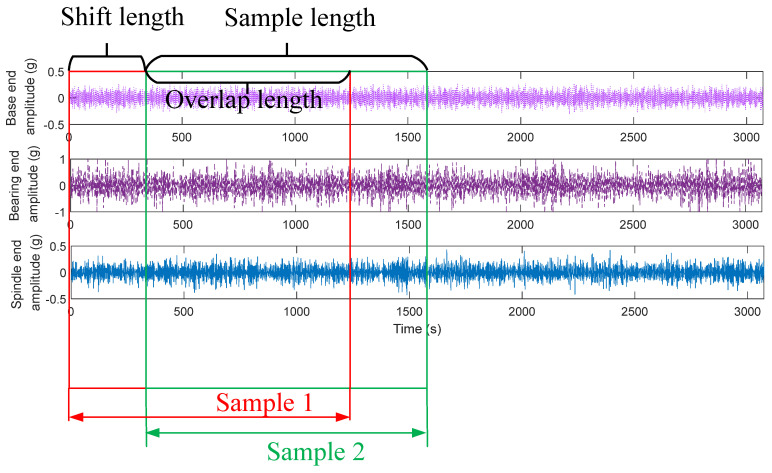
Data splitting and enhancement.

After the data acquisition was completed, data preprocessing and discretization were needed. Errors were rounded to 0.5. The spindle speed range was 1000 r/min~4000 r/min, and the range of spindle rotation error was 5 μm~14.5 μm. The rotation error data were discretized to contain a total of 20 categories. Two datasets were selected for each category. The vibration signal sampling frequency was 20 KHz, and the sampling time of each sub-dataset was 10 s. The signals in the Z-axis direction from three vibration sensors were selected as experimental data. To simulate the application in the actual industry, the data were divided according to the time order. The first 70% of the data points of each signal in order were regarded as the training data, and the last 30% of the data points were regarded as the test data. Data enhancement can improve the generalization ability of deep learning algorithms [36]. Data enhancement with overlapping samples was adopted in this article, as shown in Figure 8. For each training sample, there was an overlap of data points with the subsequent sample. The example in Figure 8 has 1024 data points per sample, and there are 704 overlapping data points.

### 4.3. Comparision Methods

To demonstrate the validity and superiority of AWResNet, we compared the AWResNet algorithm with many existing methods including LeNet, CNN, convolutional bidirectional long short-term memory (CBiLSTM), MSCNN and ResNet.

LeNet: LeNet is the classical deep learning method for image classification. The improved LeNet network structure adopted in this paper is successively as follows: Conv+ReLU layer with an output channel of 6; the maximum pooling layer area is 2×2, with a stride of 2; Conv+ReLU layer with an output channel of 16; the adaptive maximum pooling layer output size is 5×5. The LeNet model has a convolutional kernel size of 5×5.

CNN: One of the most significant differences from LeNet is that this CNN model adds the BN layer. The CNN model structure includes two Conv+BN+ReLU layers with output channels of 16 and 32; the maximum pooling layer area of 2×2, with a stride of 2; two Conv+BN+ReLU layers with output channels of 64 and 128; the adaptive maximum pooling layer output size of 4×4. The CNN model has a convolutional kernel size of 3×3.

CBiLSTM: A CNN can extract spatial correlation features from data, while bidirectional long short-term memory (BiLSTM) can extract temporal correlation features from data. According to the literature [37], CNN+BiLSTM (CBiLSTM) was adopted in comparison experiments.

MSCNN: According to the literature [23], by using different convolution kernel sizes, an MSCNN can extract multi-scale features from spindle vibration signals and achieve good results in spindle rotation error prediction. Therefore, an MSCNN was used in the comparison experiments.

ResNet: According to the literature [24], through identity mapping, ResNet can accurately predict spindle rotation error, so ResNet was used for comparison experiments.

In addition, to ensure the fairness of the comparison results, the same hyperparameters were used for the proposed model and other comparative models. We set the batch size to 64, which affected the accuracy and training speed of the model. Adam was the parameter optimization method. Momentum was an important parameter, with a value of 0.9. The learning rate had a value of 0.001. L2 regularization was employed to optimize model training. The parameter was set to 0.00001. The experimental maximum epoch was set to 100. The above parameters followed the benchmark settings for deep learning used in mechanical industry fault diagnosis in reference [37].

The performance evaluation index of the model is classification accuracy, which can be expressed as follows:

(10) $$Acc=\frac{Sum({Test}_{i}=={True}_{i})}{Sum({True}_{i})}\times 100\%$$

where $Sum({True}_{i})$ represents the number of test samples; Acc represents classification accuracy; $Sum({Test}_{i}=={True}_{i})$ indicates that the number of the predicted labels of the test sample is equal to real labels. The larger the Acc, the better the model performance.

### 4.4. Prediction Results

**Figure 9:**
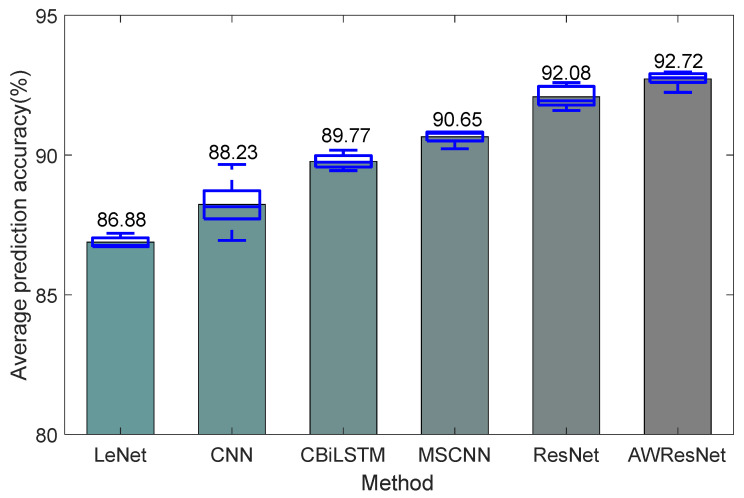
Average prediction accuracy of different methods.

Five experiments were performed for each method. The prediction accuracy and standard deviation of the five experiments are presented in Figure 9 and Table 2. The average prediction accuracy of AWResNet models was significantly better than that of other deep learning models. Compared with LeNet, CNN and BiLSTM methods, the average prediction accuracies of the AWResNet model were significantly improved by 5.84%, 4.49% and 2.95%, respectively. The above three methods have fewer convolution layers and could not extract helpful feature information from vibration data. Compared with the MSCNN, the average accuracy of the AWResNet model was improved by 2.07%. Since the MSCNN did not use identity mapping, the accuracy was lower than that of ResNet and AWResNet. The average accuracy of the AWResNet model was improved by 0.64% compared to the ResNet method. This was due to the attention weighting unit’s ability to adaptively recalibrate the importance of multi-sensor data, assigning greater weights to channel data containing more degraded information, thereby enhancing useful information and suppressing redundant information.

In addition, this paper compared the computational complexity, inference time and number of parameters of different methods, where the inference time was the time consumed by 2320 test samples on the dataset. Although LeNet, CNN and BiLSTM had relatively low computational complexity, inference time and parameters, their prediction accuracies were significantly lower than the AWResNet models. For the MSCNN, not only was the prediction accuracy lower than the AWResNet model, but the multi-scale structure also brought a large number of parametric quantities. Compared with ResNet, AWResNet brought few additional parameters, and the inference time for 2320 samples increased by 0.07 s, (translating to 0.03 ms for a single sample), and the increased inference time was negligible.

### 4.5. Confusion Matrix

**Figure 10:**
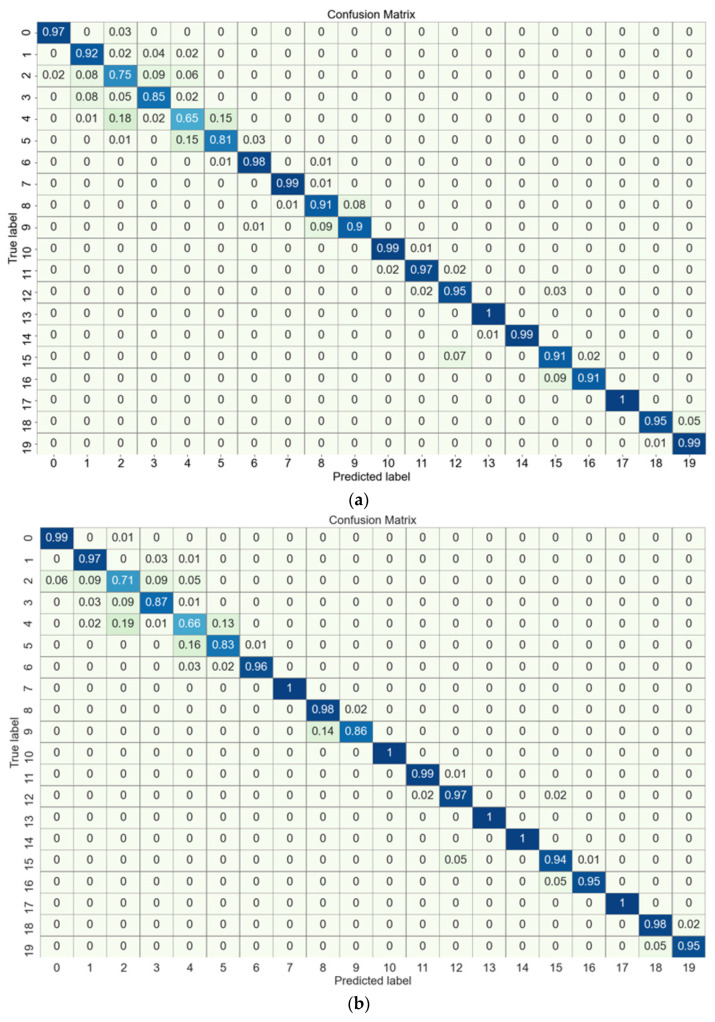
Confusion matrices of different methods: (**a**) ResNet; (**b**) AWResNet.

The confusion matrix was utilized to observe the classification accuracy of the network in each category. The confusion matrices of ResNet and AWResNet models for the classification of spindle rotation errors are shown in Figure 10. Each row represents the predicted label category, and each column represents the real label category. The data in row i and column j in the figure represent the proportion of categories in row i predicted to correspond to categories in column j. As can be seen from the figure, in 14 of the 20 classes (rotation errors were 5 μm, 5.5 μm, 6.5 μm, 7 μm, 7.5 μm, 8.5 μm, 9 μm, 10 μm, 10.5 μm, 11 μm, 12 μm, 12.5 μm, 13 μm and 14 μm), the classification accuracy of the AWResNet model was higher than the ResNet model. For two categories (11.5 μm, 13.5 μm), the classification accuracy of the AWResNet model was equal to the ResNet model. Although the accuracy of the AWResNet model was slightly lower than that of the ResNet model for rotation errors of 6 μm, 8 μm, 9.5 μm and 14.5 μm, the AWResNet predictions were generally close to the actual values. For example, 14% of the 9.5 μm samples were predicted to be 9 μm, and 5% of the 14.5 μm samples were predicted to be 14. This was due to the fact that the rotation error division spacing was too small, and the vibration signals between the current category and the neighboring categories were very similar, making it difficult for the model to extract the weak feature information. Such prediction results still represent very essential guidance for actual processing. The confusion matrix further demonstrates that AWResNet outperformed ResNet in classification in most categories.

### 4.6. Weight Visualization

**Figure 11:**
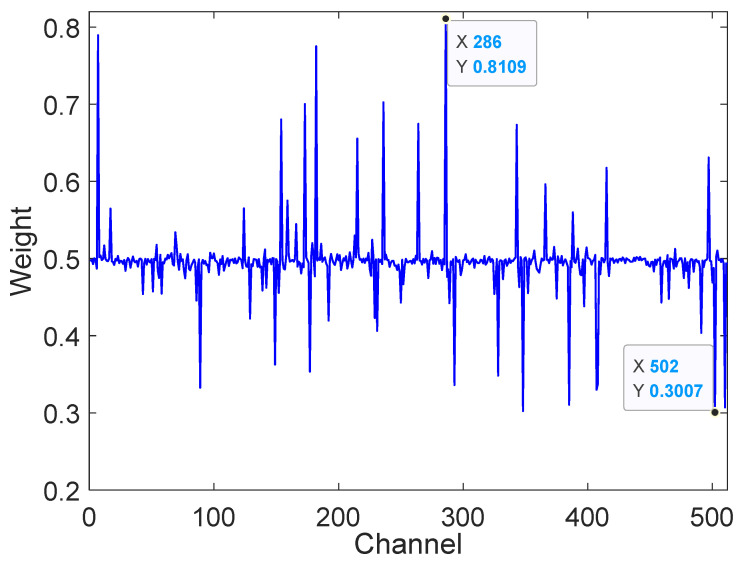
Visualization of different channel weights.

To further demonstrate that the AWResNet model can learn weights of varying sizes for different channels to recalibrate the data, thereby highlighting important information and suppressing redundant information, the weights of the last batch in the second adaptive weighting RBU4 module are visualized along 512 channels in Figure 11. The weight visualization graph indicates that different channels had different weights, and the maximum weight value was 0.8109, which appeared in channel 286. The minimum weight was 0.3007 and appeared in channel 502. As shown in Figure 11, channels 7, 173, 182, 236 and 286 all had weights more than 0.7, which corresponded to more important feature information; channels 89, 149, 177, 293, 328, 348, 385, 407, 408, 502 and 510 all had weights less than 0.4, which corresponded to relatively unimportant feature information. Attention weighting units adopted larger weights to reinforce important features, while smaller weights weakened unnecessary features. This was mainly due to differences in the data collected by the vibration sensors installed at the base end, the spindle end and the bearing end. Different rotation errors or samples are not consistently sensitive to different sensor data. Some may be more sensitive to data from the bearing end, some more sensitive to the spindle end and some more sensitive to the base end. Thus, there are differences in the importance of the data from different channels and differences in the importance of the features extracted from varying channels for the prediction of the rotation error. The weight visualization further demonstrates that attention weighting units learn different sizes of weights for different channels, emphasizing important feature information and suppressing redundant information. Thus, AWResNet can accurately extract the critical features of different rotation errors and avoid the influence of similar features.

### 4.7. Anti-Noise Experiment

In practical industrial applications, the monitoring signals collected by vibration sensors are often affected by environmental noise generated by vibration and friction, thereby reducing the quality of monitoring data. To evaluate the prediction performance of AWResNet in noisy environments, we conducted anti-noise experiments by adding Gaussian and Laplace noise signals with different signal-to-noise ratios (SNRs) to the original signal. The SNR can be expressed as follows:

(11) $$SNR=10{log}_{10}(\frac{P_{signal}}{P_{noise}})$$

where $P_{signal}$ represents the original signal power and $P_{noise}$ represents the noise signal power. In the experiment, Gaussian and Laplace noise with an SNR of 12 dB, 10 dB and 8 dB was added to the original signal. Table 3 shows that the prediction accuracy of the AWResNet model was 90.74%, 89.59% and 87.12%, respectively, when the Gaussian noise level was 12 dB, 10 dB and 8 dB. As the SNR decreased and the noise became stronger, the prediction accuracy of all models decreased, but the predictive performance of AWResNet consistently outperformed other deep learning models. In particular, compared to the ResNet model, AWResNet improved prediction accuracy by 1.41%, 1.89% and 1.11% under three noise levels. When Laplace noise was added, the prediction accuracies were 90.91%, 89.53% and 87.71% at an SNR of 12 dB, 10 dB and 8 dB, respectively. The prediction accuracy was significantly higher than that of other deep learning models. Compared to the ResNet model, it was improved by 1.73%, 1.82% and 1.77%. It can be concluded that the AWResNet model has better anti-noise robustness and stability. This advantage contributes to the practical application of the AWResNet model.

## 5. Conclusions

The existing measurement methods of spindle rotation error are usually implemented on the premise of spindle idling, which is challenging to use for real-time monitoring and real-time rotation error compensation in actual machining. The prediction method based on vibration signal does not consider multi-sensor data interaction and highlight critical sensor data. To solve this problem, a new AWResNet model for spindle rotation error prediction is proposed in this paper. The AWResNet model mainly implements the adaptive recalibration of data in different channels through the attention weighting unit embedded behind the RBU module. To evaluate the effectiveness and superiority of the AWResNet model, we carried out prediction experiments on a machine tool spindle reliability test bed and concluded the following:

(1) The results of rotation error prediction experiment show that the prediction accuracy of AWResNet model is 92.72%, which is significantly higher than other deep learning models.

(2) The confusion matrices show that the AWResNet model is more accurate than the ResNet model in 14 out of 20 categories.

(3) Weight visualization shows that the embedded attention weighting unit can learn different weights for each channel. The weight is between 0.3007 and 0.8109. AWResNet model can highlight important feature information and suppress redundant information.

(4) The anti-noise experiments indicate that the accuracy of the AWResNet model is higher than that of the ResNet model under three different noise levels of Gaussian and Laplace noise and that the AWResNet model has better robustness, stability, and more significant potential for industrial applications.

In our future research work, we will collect more rotation errors and vibration signals from different types of spindles, which will be utilized to study the generalization and transfer ability of the model.

## Figures and Tables

**Table 1:** AWResNet model structure parameters.

Layer Name	Kernel Size	Stride	Input	Output
Conv+BN+ReLU	64×7×7	2×2	3×33×33	64×17×17
Adaptive weighting RBU1 × 2	64×3×3	1×1	64×17×17	64×17×17
Adaptive weighting RBU2 × 2	128×3×3	1×1	128×17×17	128×17×17
Adaptive weighting RBU3 × 2	256×3×3	1×1	256×17×17	256×17×17
Adaptive weighting RBU4 × 2	512×3×3	1×1	512×17×17	512×17×17
GAP	-	-	512×17×17	512×1×1
FC	-	-	512×1×1	20

**Table 2:** Spindle rotation error prediction accuracy and standard deviation.

		LeNet	CNN	CBiLSTM	MSCNN	ResNet	AWResNet
Model comparison	Complexity	712.07 k	16.95 M	6.4 M	851.44 M	3.23 G	3.23 G
	Inference time	0.09 s	0.12 s	0.14 s	0.44 s	1.39 s	1.46 s
	Parameters	62.8 k	2.33 M	977.24 k	16.98 M	11.19 M	11.80 M
Experiment	First prediction	87.20	88.15	89.44	90.60	91.94	92.76
	Second prediction	86.72	89.66	90.17	90.82	92.41	92.72
	Third prediction	86.98	86.94	89.91	90.22	92.59	92.24
	Fourth prediction	86.77	88.41	89.74	90.82	91.85	92.89
	Fifth prediction	86.72	87.97	89.61	90.78	91.59	92.97
	Average and standard deviation	86.88 ± 0.21	88.23 ± 0.98	89.77 ± 0.28	90.65 ± 0.26	92.08 ± 0.41	92.72 ± 0.28

**Table 3:** Prediction accuracy in different noise environments.

Noise Type	SNR	LeNet	CNN	CBiLSTM	MSCNN	ResNet	AWResNet
Gaussian	12 dB	85.47 ± 0.98	86.99 ± 0.85	87.82 ± 0.54	89.36 ± 0.72	89.33 ± 0.71	90.74 ± 0.19
	10 dB	83.85 ± 1.26	85.73 ± 0.54	86.14 ± 0.56	88.80 ± 0.36	87.70 ± 0.28	89.59 ± 0.53
	8 dB	80.66 ± 2.44	84.53 ± 0.89	84.22 ± 0.93	87.49 ± 0.95	86.01 ± 0.66	87.12 ± 0.81
Laplace	12 dB	85.54 ± 0.82	86.81 ± 0.44	88.34 ± 0.64	89.70 ± 0.38	89.18 ± 1.02	90.91 ± 0.21
	10 dB	84.16 ± 0.96	86.32 ± 0.54	86.12 ± 0.64	88.72 ± 0.62	87.71 ± 0.53	89.53 ± 0.22
	8 dB	79.69 ± 3.08	85.01 ± 1.04	85.10 ± 0.85	87.39 ± 0.75	85.94 ± 0.52	87.71 ± 0.67

## Data Availability

The data presented in this study are available on request from the corresponding author.

## References

[1] Guo Z. (2019). Precision design and measurement method of large precision rotary body rotary. Manuf. Technol. Mach. Tool.

[2] Zuo Y., Wang H., Wu G., Gu Y., Qiao W. (2019). Research on remote state monitoring and intelligent maintenance system of CNC machine tools. J. Eng..

[3] Lee J., Tsai-Lin C., Lee Y., Liu M., Chen L. (2017). Fully automatic CNC machining production system. MATEC Web Conf..

[4] Castro H. (2008). A method for evaluating spindle rotation errors of machine tools using a laser interferometer. Measurement.

[5] Liu F., Liang L., Xu G., Hou C., Liu D. (2020). Four-point method in the measurement and separation of spindle rotation error. IEEE/ASME Trans. Mechatron..

[6] Wang L., Zhang B., Wu J. (2018). Rotation accuracy evaluation of electric spindle based on least square method. Manuf. Technol. Mach. Tool.

[7] Wang L., Zhao Q., Zhang B. (2018). Accuracy of an electric spindle. J. Tsinghua Univ. (Sci. Technol.).

[8] Anandan K.P., Ozdoganlar O.B. (2016). A multi-orientation error separation technique for spindle metrology of miniature ultra-high-speed spindles. Precis. Eng..

[9] Karacay T., Akturk N. (2008). Vibrations of a grinding spindle supported by angular contact ball bearings. Proc. Inst. Mech. Eng. Part K J. Multi-Body Dyn..

[10] Kang T., Cao H. (2020). Dynamic prediction method for machine tool spindle rotational accuracy under cutting condition. J. Mech. Eng..

[11] Bai F. (2020). Research on Deterioration and Sustainability Evaluation of Rotation Accuracy of Motorized Spindle. Master’s Thesis.

[12] Liang J., Wang L., Yu G., Wu J., Wang D., Lin S. (2024). Indirect Prediction of Spindle Rotation Error Through Vibration Signal Based On Supervised Local Mean Decomposition Filter Fusion and Bi-LSTM Classification Network. ASCE-ASME J. Risk Uncert. Engrg. Sys. Part B Mech. Engrg..

[13] Chen D., Wu J., Wang L., Liang J., Gong X., Kong W. (2020). A method for predicting spindle rotation accuracy using vibration. Sci. Sin. Technol..

[14] Urbanek J., Barszcz T., Strączkiewicz M., Jablonski A. (2017). Normalization of vibration signals generated under highly varying speed and load with application to signal separation. Mech. Syst. Signal Proc..

[15] Jia Y., Li G., Dong X., He K. (2021). A novel denoising method for vibration signal of hob spindle based on EEMD and grey theory. Measurement.

[16] Liang J., Wang L., Wu J., Liu Z., Yu G. (2021). Prediction of spindle rotation error through vibration signal based on Bi-LSTM classification network. IOP Conference Series: Materials Science and Engineering.

[17] Gao T., Yang J., Jiang S. (2022). A Novel Fault Detection Model Based on Vector Quantization Sparse Autoencoder for Nonlinear Complex Systems. IEEE Trans. Ind. Inform..

[18] Gao T., Yang J., Tang Q. (2024). A multi-source domain information fusion network for rotating machinery fault diagnosis under variable operating conditions. Inf. Fusion..

[19] Yang J., Gao T., Yan G., Yang C., Li G. (2023). A fault location method based on ensemble complex spatio-temporal attention network for complex systems under fluctuating operating conditions. Appl. Soft Comput..

[20] Song L., Wu J., Wang L., Chen G., Shi Y., Liu Z. (2023). Remaining Useful Life Prediction of Rolling Bearings Based on Multi-Scale Attention Residual Network. Entropy.

[21] Zhou J., Qin Y., Chen D., Liu F., Qian Q. (2022). Remaining useful life prediction of bearings by a new reinforced memory GRU network. Adv. Eng. Inform..

[22] Jiang G., Zhou W., Chen Q., He Q., Xie P. (2022). Dual residual attention network for remaining useful life prediction of bearings. Measurement.

[23] Song L., Wang L., Wu J., Guan L., Liu Z. (2022). Reliability analysis based on cyber-physical system and digital twin. J. Jilin Univ. (Eng. Technol. Ed.).

[24] Song L., Wang L., Wu J., Liang J., Liu Z. (2021). Integrating physics and data driven cyber-physical system for condition monitoring of critical transmission components in smart production line. Appl. Sci..

[25] Qu H., Zhang H., Yan W., Ma F. (2021). Energy Efficiency Level Prediction of CNC Milling Machine Based on Improved Convolution Neural Network. Mach. Tool Hydraul..

[26] Krizhevsky A., Sutskever I., Hinton G.E. (2017). ImageNet classification with deep convolutional neural networks. Commun. ACM.

[27] Zhao B., Zhang X., Li H., Yang Z. (2020). Intelligent fault diagnosis of rolling bearings based on normalized CNN considering data imbalance and variable working conditions. Knowl.-Based Syst..

[28] Zhao B., Zhang X., Zhan Z., Pang S. (2020). Deep multi-scale convolutional transfer learning network: A novel method for intelligent fault diagnosis of rolling bearings under variable working conditions and domains. Neurocomputing.

[29] Wang B., Lei Y., Yan T., Li N., Guo L. (2020). Recurrent convolutional neural network: A new framework for remaining useful life prediction of machinery. Neurocomputing.

[30] Wang B., Lei Y., Li N., Yan T. (2019). Deep separable convolutional network for remaining useful life prediction of machinery. Mech. Syst. Signal Proc..

[31] Jing L., Zhao M., Li P., Xu X. (2017). A convolutional neural network based feature learning and fault diagnosis method for the condition monitoring of gearbox. Measurement.

[32] He K., Zhang X., Ren S., Sun J. Deep residual learning for image recognition. Proceedings of the IEEE Conference on Computer Vision and Pattern Recognition.

[33] Ioffe S., Szegedy C. Batch normalization: Accelerating deep network training by reducing internal covariate shift. Proceedings of the 32nd International Conference on Machine Learning (ICML 2015).

[34] Hu J., Shen L., Sun G. Squeeze-and-excitation networks. Proceedings of the IEEE Conference on Computer Vision and Pattern Recognition (CVPR).

[35] Chen D., Wu J., Zhang B., Wang L., Liang J. (2018). Load spectrum compilation for machining center spindles based on S-shaped specimens. J. Tsinghua Univ. (Sci. Technol.).

[36] Lecun Y., Bengio Y., Hinton G. (2015). Deep learning. Nature.

[37] Zhao Z.B., Li T.F., Wu J.Y., Sun C., Wang S.B., Yan R.Q., Chen X.F. (2020). Deep learning algorithms for rotating machinery intelligent diagnosis: An open source benchmark study. ISA Trans..

